# Recurrent IDH-mutant astrocytoma WHO grade 4 diagnosed during pregnancy: case report with literature review

**DOI:** 10.3389/fonc.2026.1804727

**Published:** 2026-05-28

**Authors:** Muratbek A. Tleubergenov, Altynay Bakbayeva, Daniyar K. Zhamoldin, Aidos Doskaliyev, Ulday M. Urazova, Aigul D. Tolepbergenova, Aisa Z. Nurpeisov, Zhanat T. Takenov, Serik Akshulakov

**Affiliations:** 1 National center of Neurosurgery Joint - Stock company (NCJSC) “Astana Medical University”, Astana, Kazakhstan; 2 Nazarbayev University School of Medicine, Astana, Kazakhstan; 3 Department of Brain Neurosurgery, Joint-Stock Company (JSC) “National Centre for Neurosurgery”, Astana, Kazakhstan; 4 Chief Executive Officer (CEO), Joint-Stock Company (JSC) “National Centre for Neurosurgery”, Astana, Kazakhstan

**Keywords:** CDKN2A/B deletion, CNS tumors in pregnancy, high-grade glioma, IDH1-mutant astrocytoma, pregnancy, simultaneous cesarean and craniotomy, WHO grade 4 glioma

## Abstract

High-grade gliomas during pregnancy are exceedingly rare and pose complex clinical challenges. Molecular features such as IDH mutations and CDKN2A/B deletions have become central to diagnosis and prognostication in the 2021 WHO classification of CNS tumors. We report the case of a 31-year-old pregnant woman diagnosed at 24 + 5 weeks of gestation with an IDH1-mutant astrocytoma, WHO Grade 4, harboring a heterozygous CDKN2A/B deletion. She initially presented with progressive neurological symptoms and underwent emergency craniotomy with subtotal tumor resection. Despite early neurosurgical intervention and temporary clinical stabilization, the tumor recurred rapidly within five weeks, prompting a simultaneous cesarean section and re-craniotomy at 31 weeks’ gestation. Both mother and infant survived the perioperative period without complications. However, the patient passed away within two years of diagnosis, highlighting the tumor’s aggressive course. This case illustrates the poor prognostic implications of CDKN2A/B loss, even in tumors with a favorable IDH mutation status. The molecular profile—especially the presence of any CDKN2A/B deletion—may define a more aggressive subtype of IDH-mutant astrocytoma. Timely neurosurgical intervention, multidisciplinary care, and integrated obstetric-neurosurgical strategies are crucial. There is a pressing need for clinical guidelines addressing high-grade glioma management in pregnancy, particularly in the era of molecular tumor classification.

## 1 Introduction

Astrocytoma, IDH-mutant, CNS WHO Grade 4 is a highly malignant form of a primary brain tumor, recently reclassified under the 2021 WHO classification of central nervous system tumors. Arising from astrocytes, it represents a diffusely infiltrative glioma characterized by a mutation in the isocitrate dehydrogenase (IDH) gene, most commonly IDH1, and often accompanied by additional genetic alterations such as homozygous or heterozygous deletion of CDKN2A/2B. This tumor, previously termed “Glioblastoma, IDH-mutant”, typically arises in younger adults and genetically carries a better prognosis compared to IDH-wildtype variants (1).

The occurrence of gliomas during pregnancy is extremely rare, with an estimated incidence of primary brain tumors between 2.6 and 15 per 100,000 pregnancies worldwide (2). High-grade gliomas such as Grade 4 astrocytomas are particularly uncommon. Although pregnancy does not appear to increase the incidence of glioma, several studies suggest it may accelerate tumor progression due to hormonal, hemodynamic, and immunological changes, all of which can contribute to increased tumor growth, earlier symptom onset, and increased seizure frequency (2, 3).

The management of high-grade gliomas during pregnancy poses significant clinical and ethical challenges. Currently, no standardized treatment guidelines exist, especially for tumors diagnosed during pregnancy. Critical unanswered questions include optimal monitoring strategies, safe timing of interventions, and the most appropriate mode and timing of delivery. Multidisciplinary coordination involving neurosurgery, obstetrics, neuro-oncology, and neonatology is essential to optimize outcomes for both mother and fetus.

This report describes a rare case of an IDH-mutant Grade 4 astrocytoma with heterozygous *CDKN2A/B* deletion diagnosed during the second trimester, with aggressive progression and recurrence necessitating emergency neurosurgical intervention and a simultaneous cesarean section with tumor resection. Through this case, we aim to contribute to the limited literature on high-grade glioma management in pregnancy and highlight key considerations for clinical decision-making in similar scenarios.

## 2 Case

A 31-year-old pregnant woman (gravida 4, para 2) at 24 weeks and 5 days of gestation presented with a one-month history of progressive neurological symptoms, including headaches, dizziness, visual and speech disturbances, drowsiness, nausea, vomiting, generalized weakness, poor appetite, and a 3 kg weight loss. Her past medical history included primary hypothyroidism, chronic pyelonephritis (in remission), and angiopathy. As symptoms worsened, she sought neurological evaluation, which led to rapid clinical deterioration and emergency ICU admission at the regional hospital in early July 2023. From there, she was transported by Sanaviation to the National Centre for Neurosurgery in Astana, Kazakhstan, for further evaluation and management.

## 3 Investigation

The patient demonstrated a Glasgow Coma Scale score of 13–14, indicating mildly impaired consciousness. Insight and awareness of her condition were diminished. She provided correct answers to questions, though often only after repeated prompts, and followed instructions with noticeable mental fatigue. Cognitive assessment revealed mildly impaired calculation ability and reduced memory function. Her Karnofsky Performance Status was estimated at 40%, reflecting significant functional limitation. Cranial nerve examination was unremarkable except for bilateral optic nerve involvement, consistent with amblyopia in both eyes. The Romberg test showed instability, and coordination tests revealed dysmetria. All other neurological findings were within normal limits.

MRI findings revealed a space-occupying lesion in the left frontal lobe of the brain, measuring approximately 6.0 × 5.8 × 6.5 cm, associated with a 15 mm midline shift as shown in **Figure 1**. The heterogeneous mass exhibited features of central necrosis and was surrounded by moderate peritumoral edema.

**Figure 1:**
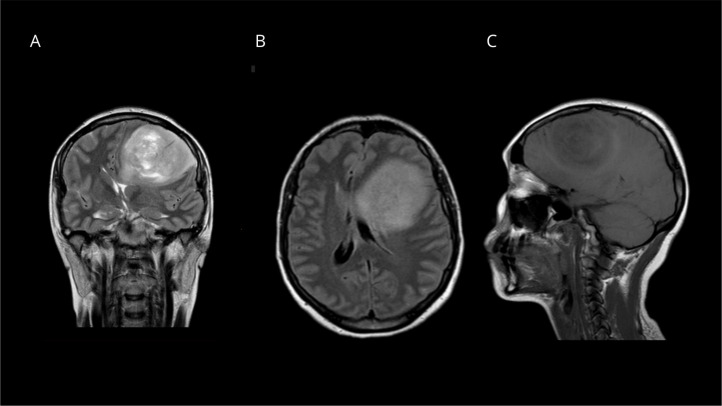
Pre-operative MRI. MRI demonstrates a large left frontal lobe lesion with heterogeneous signal characteristics. On T2-weighted imaging (**(A)**, coronal), the mass appears hyperintense, with central necrosis and extensive peritumoral edema, contributing to a significant mass effect and midline shift. On T1-weighted imaging (**(B)**, axial and **(C)**, sagittal), the lesion is hypointense to isointense, with central hypointensity reflecting necrosis. There is notable effacement of the left lateral ventricle, midline shift **(B)**, and distortion of anterior cranial fossa structures **(C)**.

A national telemedicine consultation was convened, during which a multidisciplinary team recommended urgent transfer to a specialized National Centre for Neurosurgery for further evaluation and management.

Given the tumor’s aggressive growth, a multidisciplinary team—including obstetricians, neurosurgeons, anesthetists, an ophthalmologist, a neurologist, and midwives—convened to plan its surgical removal, coordinate perioperative care, and establish protocols for managing potential emergencies.

## 4 Differential diagnosis

Based on the MRI findings, the lesion located in the left frontal lobe measures approximately 6.0 × 5.8 × 6.5 cm and is associated with a significant 15 mm midline shift. The mass demonstrates heterogeneous signal intensity, central necrosis, and is surrounded by moderate peritumoral edema—hallmarks of a high-grade infiltrative process. The constellation of imaging features—rapid growth, mass effect with midline shift, central necrosis, and perilesional vasogenic edema—is characteristic of a high-grade glioma, particularly glioblastoma or IDH-mutant astrocytoma, WHO Grade 3 or 4, depending on histological examination. Given the size, location, and radiological profile, no other diagnosis adequately accounts for the lesion’s aggressive behavior and imaging morphology. Therefore, high-grade glioma remains the singular, most appropriate diagnosis in this clinical context.

## 5 Management and treatment

### 5.1 Operative technique (first surgery)

A craniotomy in the projection of the left frontal-parietal-temporal region was performed under general anesthesia with intraoperative fetal monitoring by using Holter cardiotocography (CTG). The patient’s head was positioned and fixed in a Mayfield clamp with a 30-degree turn to the right. Using intraoperative neuronavigation, the borders of the tumor were localized.

A 15 cm arcuate projective skin incision was made, and a 6.0 × 5.0 cm bone flap was elevated. The dura mater appeared tense, bulging, and without visible pulsation. Upon dural opening, the underlying cortex was exposed and appeared markedly edematous. At a depth of ~0.5 cm, a dense, gray-brown infiltrative tumor was identified in the anterior frontal lobe (~6.0 × 5.0 cm) with hypervascularity and necrotic tissue.

Microsurgical tumor removal was performed using a Pentero operating microscope (8–12× magnification) and standard microsurgical instruments. Resection margins were guided by neuronavigation. The resection cavity measured 6.5 × 6.0 × 5.5 cm. The tumor was removed subtotally due to its infiltrative nature and involvement of eloquent brain regions. Hemostasis was achieved with coagulation and Surgicel.

Duraclosure was performed using autologous periosteum, the bone flap was replaced, layered closure was done, and active drainage was placed for 24 hours.

### 5.2 Histopathological and molecular evaluation

Histology showed a highly cellular glial neoplasm with pleomorphism, vascular proliferation, and pseudopalisading necrosis, consistent with a high-grade glioma. Molecular testing revealed an IDH1 p.R132H mutation and a heterozygous CDKN2A/2B deletion, confirming the diagnosis of IDH-mutant astrocytoma, WHO Grade 4. Additional molecular analyzes, including assessment of TERT, EGFR, and MYC, were performed, and no mutations or amplifications were identified.

### 5.3 Postoperative imaging and fetal assessment

Postoperatively, the patient was admitted to the intensive care unit (ICU), where she received comprehensive care, including antibiotic prophylaxis, antiedema therapy, analgesia, gastroprotective agents, hormonal therapy, intravenous fluids, replacement therapy, and symptomatic management, along with continuous clinical and neurological monitoring.

On postoperative day one, the patient remained in the ICU, was mobilized, and demonstrated clinical stability. By postoperative day two, her condition remained stable and without complications, allowing for her transfer to the specialized neurosurgical ward. Fetal assessment confirmed a normal status throughout the postoperative period.

Postoperative brain MRI on the 5th day revealed a resection cavity filled with CSF-like fluid, perifocal edema, and signs of ventricular straightening. The patient’s neurological deficits improved following surgery. A fetal ultrasound performed four days later confirmed a 25-week + 1-day breech pregnancy without signs of acute fetal distress.

The patient was discharged in stable condition with recommendations for adjuvant chemoradiotherapy after consultation with a neuro-oncologist.

### 5.4 Clinical progression and recurrence

Approximately one month later, at 31 weeks of gestation, the patient developed worsening contralateral central hemiparesis on the ⅗ MRC scale and motor aphasia. The progression of clinical manifestations is summarized in **Table 1**. MRI revealed a large recurrent tumor (8.3 × 8.2 × 6.7 cm) involving the frontal and cingulate gyri, with peritumoral edema, ventricular compression, and 1.6 cm midline shift. Due to rapid tumor progression and significant maternal risk, including a high likelihood of maternal mortality, thromboembolic events, sepsis, and hemorrhage, a multidisciplinary team recommended early delivery combined with tumor resection. To reduce anesthesia-related risks and avoid multiple surgeries, a single-session approach was selected, and the patient underwent a simultaneous cesarean section and neurosurgical tumor resection.

**Table 1:** Timeline.

5 July 2023	Initial consultation with a neurologist and hospitalization to the ICU
6 July 2023	Initial brain MRI (pre-surgery) revealed a large tumor mass
12 July 2023	First tumor removal
13 July 2023	Postoperative CT and histological results
15 July 2023	Fetal ultrasound confirms 25 + 1 weeks of gestation with breech position
1 September 2023	Follow-up MRI reveals recurrence of the tumor
7 September 2023	Start of second hospitalization
11 September 2023	Simultaneous cesarean section and removal of progressive tumor growth
12 September 2023	Postoperative Day 1 CT scan
15 September 2023	Postoperative Day 4 CT scan
25 September 2023	Discharge from the second hospitalization

### 5.5 Second surgery: simultaneous cesarean section and tumor resection

The procedure was performed in two stages under general anesthesia.

#### 5.5.1 Stage 1 – cesarean section

A lower midline laparotomy was carried out, and a live male infant was delivered in breech presentation, weighing 1650 grams with an Apgar score of 7 at one minute. The uterus was sutured in two layers, and hemostasis was secured. The abdominal cavity was inspected and closed in layers without complications. The newborn was handed over to the neonatal team for further care.

#### 5.5.2 Stage 2 – neurosurgical tumor resection

Following delivery, the patient’s head was positioned in a Mayfield clamp with a 30-degree rightward rotation. A left fronto-temporo-parietal craniotomy was performed along the previous surgical scar. A 6 × 6 × 5 cm bone flap was elevated. Upon opening the dura mater, tense, bulging, and non-pulsatile, a markedly edematous cortex was visualized.

Using intraoperative neuronavigation, a gray-brown, hypervascular, infiltrative tumor was identified in the frontal lobe (8.3 × 8.2 × 6.7 cm). Microsurgical subtotal resection was performed using a Karl Zeiss operating microscope (8–12× magnification), with tumor extension noted into the basal ganglia and corpus callosum. Due to the infiltrative nature and proximity to eloquent brain regions, only subtotal resection was feasible. The tumor resection cavity measured approximately 7.5 × 7.0 × 6.0 cm. Hemostasis was achieved, dura closure was performed, the bone flap was replaced, and an active drain was inserted for 24 h.

### 5.6 Postoperative course

The infant was admitted to the neonatal intensive care unit (NICU) for routine monitoring and supportive care and was subsequently discharged in stable condition. Postoperatively, the patient was managed in the intensive care unit (ICU), where she received comprehensive treatment, including antibiotic prophylaxis, antiedema therapy, analgesia, gastroprotective agents, hormonal therapy, intravenous fluids, replacement therapy, and symptomatic management, with continuous clinical and neurological monitoring.

In the immediate postoperative period, the patient demonstrated measurable neurological improvement. Speech output increased, motor aphasia was reduced, and right-sided hemiparesis improved from grade 3 to grade 4 on the Medical Research Council (MRC) scale, indicating partial restoration of motor function and early signs of neurological stabilization following tumor resection.

A CT scan on postoperative day 1 showed a resection cavity filled with cerebrospinal fluid, peripheral hemorrhagic components, persistent cerebral edema, and a 10 mm midline shift, along with compression of the lateral and third ventricles and narrowing of the extracerebral CSF spaces. By postoperative day 4, follow-up CT revealed ongoing mass effect, a 9 mm midline shift, ventricular deformation, and partial herniation of the left frontal lobe through the craniotomy site.

The patient was discharged in stable condition with recommendations for adjuvant chemoradiotherapy as part of her ongoing oncological management.

## 6 Discussion

The diagnosis and management of high-grade gliomas during pregnancy are particularly rare and complex, posing both clinical and ethical challenges for the medical team. This case stands out due to the diagnosis of an IDH-mutant WHO Grade 4 astrocytoma during the second trimester, followed by tumor recurrence and a simultaneous cesarean section and repeat craniotomy. While the co-occurrence of pregnancy and glioma is documented, cases involving IDH-mutant Grade 4 astrocytomas with such an aggressive and recurrent course are exceedingly uncommon.

Due to the lack of established guidelines, particularly regarding the timing and safety of neurosurgical interventions during pregnancy, treatment decisions must be individualized and guided by a multidisciplinary team. The primary goal in this case was to preserve both the pregnancy and the woman’s health. Given the individual approach and the pregnant woman’s preference to maintain the pregnancy, the decision to proceed with surgery was made. This decision was further supported by rapid neurological deterioration, mass effect seen on imaging, and the potential for further clinical compromise, making early neurosurgical resection the preferred option. This approach aligns with previously reported cases (4, 5), where maternal neurological status was prioritized, and the decision to proceed with surgery over abortion was made to protect both maternal and fetal outcomes. Moreover, the second trimester is considered the optimal time for surgery, as both the literature review and the mother’s neurological status suggest that it balances maternal and fetal safety, considering the increased fetal vulnerability in the first trimester and the higher risk of intraoperative hemorrhage in the third trimester (2).

As reported by Lally et al. (6, the average time to first recurrence for IDH-mutant astrocytomas was 531 days (17.5 months; 95% CI: 9.5–25.4), with a median of 299 days (9.8 months; IQR: 238–820). While specific statistics on the recurrence rates of high-grade gliomas during pregnancy are limited, several studies have observed tumor progression and clinical deterioration during pregnancy (2). Recurrent high-grade astrocytomas often continue to progress despite initial treatment. Management usually involves surgical resection, which can extend life but is limited by risks, especially in areas with high vascularization. Reirradiation is rarely used due to the risk of necrosis in healthy brain tissue. Other treatments, like radiotherapy and radiosurgery, have variable effectiveness (7). In the case of the pregnant woman at 31 weeks, surgery was chosen due to rapid neurological decline and tumor mass effect. A simultaneous cesarean section and craniotomy minimized anesthesia exposure, reduced labor stress, and ensured an optimal surgical field. This approach balanced both maternal and fetal safety, avoiding the risks of multiple surgeries.

In this case, the diagnosis of an IDH1-mutant astrocytoma, WHO Grade 4, would typically suggest a more indolent clinical course and better prognosis, as IDH-mutant gliomas are generally slower-growing and respond more favorably to standard therapies such as radiotherapy and chemotherapy (8). The IDH1 mutation results in the production of the oncometabolite 2-hydroxyglutarate, which alters epigenetic regulation and contributes to tumor initiation, but is also associated with reduced glycolysis and slower proliferation (9). However, our patient’s disease course was notably aggressive, with rapid progression and recurrence during pregnancy, necessitating two craniotomies within a short time frame. This clinical trajectory may be partly explained by the presence of a deletion of the CDKN2A/2B genes, which is increasingly recognized as a key molecular marker of poor prognosis in IDH-mutant gliomas (10). CDKN2A/2B encodes tumor suppressor proteins (p16^INK4a and p14^ARF) that regulate the cell cycle via the p53 and Rb pathways (11). Deletion of these genes is associated with increased cellular proliferation, treatment resistance, and higher risk of malignant progression—even in tumors with otherwise favorable molecular features (12). While the literature predominantly focuses on homozygous deletions as a poor prognostic marker, recent large-scale analysis of nearly 1,000 IDH-mutant astrocytomas demonstrated that even heterozygous CDKN2A/B loss significantly reduces survival. In WHO Grade 4 tumors, overall survival for patients with hemizygous and homozygous deletions was similarly poor at 31.9 and 32.5 months, respectively (13). This highlights that any loss of CDKN2A/B may denote a biologically aggressive tumor phenotype, underscoring the potential need to consider both allelic status and co-occurring genomic alterations when assessing prognosis in IDH-mutant gliomas.

In the current WHO classification, astrocytomas are graded from 2 to 4 based on histologic and molecular features. Grade 4 IDH-mutant astrocytoma is defined not only by histological hallmarks of malignancy (e.g., necrosis, microvascular proliferation) but also by certain molecular alterations, including CDKN2A/B loss, that confer a high-risk profile (14). Despite the IDH mutation being a favorable factor, the co-occurrence of high-grade features leads to aggressive behavior and poor outcomes (15).

Overall, the co-occurrence of an IDH1 mutation with CDKN2A/2B loss has been proposed as a defining feature of molecularly aggressive astrocytoma, Grade 4, within the updated WHO classification (10). Therefore, while the IDH1 mutation alone might have suggested a relatively favorable course, the presence of CDKN2A/2B deletion likely contributed to the unexpectedly rapid progression observed in this patient.

This case highlights the importance of early neuroimaging in pregnant patients with persistent neurological symptoms and shows that neurosurgical intervention, when clearly indicated, can be safely performed during pregnancy with careful multidisciplinary planning. The successful outcome of a combined cesarean section and tumor resection suggests that this approach may be viable in select high-risk cases. This experience calls for more structured clinical guidelines and collaborative care models for managing CNS tumors during pregnancy.

Additionally, the presence of an IDH mutation and CDKN2A/B deletion emphasizes the aggressive nature of grade 4 astrocytomas. While IDH mutations are typically associated with better outcomes in lower-grade gliomas, their benefit is limited in grade 4 tumors, especially when coupled with CDKN2A/B deletions (16). These genetic factors underline the need for personalized treatment and further research to understand their impact on treatment response and prognosis, particularly in pregnant patients.

### 6.1 Limitations

This study has several limitations. First, as a single case report, the findings are inherently limited in generalizability and cannot be extrapolated to broader patient populations. Second, the absence of comprehensive histopathological imaging and detailed molecular profiling data restricts the depth of pathological correlation. Third, certain molecular analyzes, including extended genomic profiling, were not available, which may limit the full characterization of the tumor’s biological behavior.

In addition, the influence of pregnancy on tumor progression remains difficult to interpret due to the lack of robust comparative data and the complex interplay of hormonal and physiological factors. Finally, long-term follow-up data, particularly regarding treatment response and neurodevelopmental outcomes of the infant, remain limited.

Despite these limitations, this case provides valuable insight into the clinical course and management challenges of high-grade IDH-mutant astrocytoma during pregnancy.

## 7 Conclusion

This case highlights the profound impact of molecular profiling—particularly the presence of IDH1 mutation and CDKN2A/B deletion—on the clinical course of astrocytoma during pregnancy. While IDH mutations in gliomas are generally associated with a more favorable prognosis, their protective effect may be significantly diminished in the presence of CDKN2A/B loss, a marker now recognized as defining WHO Grade 4 astrocytomas. The heterozygous deletion of CDKN2A/B observed in this patient likely contributed to the tumor’s unexpectedly rapid progression and early recurrence, even within the typically less aggressive IDH-mutant molecular subtype.

Despite careful surgical planning, timely interventions, and multidisciplinary coordination, the tumor’s aggressive biology overrode many favorable clinical variables—including young maternal age, second-trimester timing of intervention, and early neurosurgical action. The outcome underscores the need to treat IDH-mutant astrocytomas with CDKN2A/B deletion as a distinct, high-risk subgroup requiring closer monitoring, personalized treatment strategies, and potentially novel therapeutic approaches.

## Data Availability

The original contributions presented in the study are included in the article/supplementary material. Further inquiries can be directed to the corresponding author.
